# Differences in brand versus generic esmolol in the treatment of perioperative supraventricular tachycardia and hypertension: A pilot study

**DOI:** 10.1177/2050312120962338

**Published:** 2020-09-30

**Authors:** Diamanto Aretha, Panagiotis Kiekkas, Nektarios Sioulas, Fotini Fligou

**Affiliations:** 1 Department of Anesthesiology and Intensive Care Medicine, School of Medicine, University Hospital of Patras, Patras, Greece; 2 Western Greece University of Applied Sciences, Patras, Greece; 3 General Hospital of Pyrgos, Pyrgos, Greece; 4 Department of Anesthesiology and Intensive Care Medicine, School of Medicine, University Hospital of Patras, Rion, Patras, Greece

**Keywords:** Esmolol, b-blocker, brand drug, generic drug, tachycardia, hypertension, perioperative

## Abstract

**Background:**

Once a patent expires, generic analogue drugs are alternatives to brand name drugs. Because bioequivalence/biodistribution problems have been reported for many generic analogue drugs, we prospectively evaluated 31 patients to reveal the differences in the doses used and the efficacy and adverse events of two different intravenous esmolol formulations.

**Methods:**

This was a prospective observational pilot study. Our aim was to reveal the possible differences in the required doses between two different formulations (brand name drug vs generic analogue drug) of intravenous esmolol in beats per minute, systolic blood pressure, diastolic blood pressure and mean arterial pressure in intra- and postoperative patients with supraventricular tachycardia and hypertension. The patients were categorised into two groups according to the medication they received (brand name drug or generic analogue drug).

**Results:**

Esmolol was given to 31 patients (16 generic analogue drug and 15 brand name drug). Although there was a statistically significant difference in bolus (mg/kg) and continued (mg/kg/h) drug dose used (brand name drug/generic analogue drug, mean (standard deviation), 0.3 (0.1) vs 0.38 (0.1), p = 0.03 for bolus dose, and 0.22 (0.09) vs 0.29 (0.08) for continued dose at 10 min (p = 0.03), 0.19 (0.06) vs 0.24 (0.05) at 20 min (p = 0.01) and 0.14 (0.05) vs 0.18 (0.05) at 30 min (p = 0.02)), there were no time-related statistical significant differences in the reduction rates of the two drugs (p = 0.47). There were no time-related statistically significant differences between the two groups in systolic blood pressure, diastolic blood pressure, mean arterial pressure and beats per minute, nor in their adverse events.

**Conclusion:**

In this pilot study, smaller doses were given for controlling the patient’s haemodynamics when a brand name drug was used. Because there were no significant time-related differences in the reduction rates of the two drugs nor in any haemodynamic differences between the two groups, optimal titration of the drug used could effectively control the patient’s haemodynamics. The adverse events were also similar in both groups.

## Introduction

Because they are generally less expensive, generic analogue drugs (GADs) are alternatives to brand name drugs (BNDs) once the patent has expired. The European Economic Area and Greece define two medicinal products as equivalent if their bioavailabilities after administration of the same molar dose are similar to such an extent that their effects, in terms of both their efficacy and safety, will be essentially the same. This applies if the 90% confidence intervals (90% CIs) of the ratios for AUC_0–t_ and C_max_ between the two preparations lie in the range of 80%–125%. Although GADs and BNDs are considered clinically equivalent and used interchangeably once approved by health system authorities, many questions have been raised for many drug classes about the clinical equivalence of GADs versus their BNDs.^1–10^

The bioequivalence of brand and generic products usually refers to oral medications, where the majority of the concerns surround the rate and extent of absorption of these products.^5^ For intravenous products, this concern is usually not likely to be an issue with maximum concentrations, where the time to the maximum concentration is considered to be similar. However, biodistribution differences following intravenous dosing of different regimens constitute a possible issue.^11–13^

Because there are anecdotal reports that generic drugs are less effective than their branded counterparts^14–16^ and considering that esmolol is a cardiovascular drug routinely used in the perioperative setting, the aim of our study was to evaluate the possible differences in efficacy and adverse events in esmolol GAD versus BND after the generic drug’s introduction. To the best of our knowledge, there are no publications that evaluate possible differences between the two preparations. We hypothesised that there would be no differences between the two formulations in the doses required and/or in their effectiveness when controlling hypertension and heart rate in the perioperative period (null hypothesis).

### Methods

**Figure 1.**
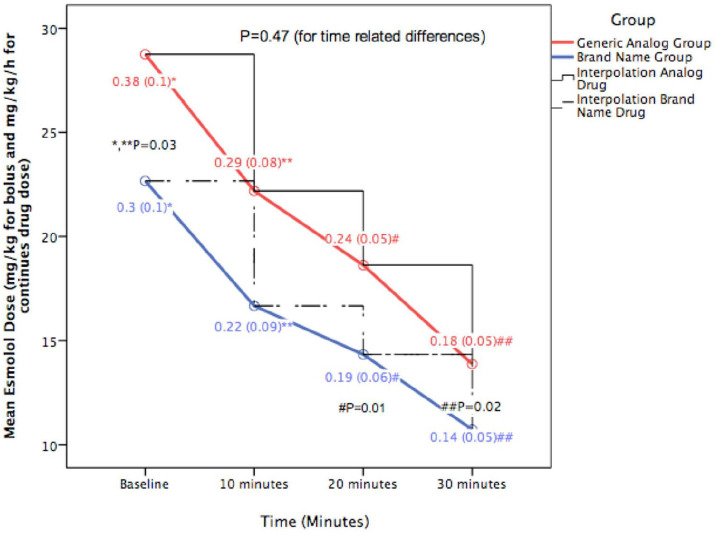
Mean time–related differences in esmolol dose (mg/kg for baseline and mg/kg/h for continues doses at baseline, 10, 20 and 30 min) between the two groups (generic analogue vs brand name group). Data are presented as mean (SD).

Considering that the optimal titration of a GAD could be a solution to the possible discrepancies in biodistribution and because of the limited data available in the literature, we conducted a pilot prospective observational study in the perioperative setting of a secondary hospital (General Hospital of Pyrgos, Greece). The aim of the current pilot study was to reveal the possible differences in the required doses between two different formulations (BND vs GAD) of intravenous (IV) esmolol in controlling beats per minute (BPM), systolic blood pressure (SBP), diastolic blood pressure (DBP) and mean blood pressure (MBP) in intra- and postoperative patients suffering from supraventricular tachycardia (SVT) and hypertension. Any statistically significant differences in the doses needed and/or in the patient’s haemodynamics between the BND and GAD were concerned a pilot success criterion. The dose titration depended on the anaesthesiologist on duty, and the goal was a difference between 20% and 30% from the patient’s baseline measures, enhancing SBP <140 mm Hg and BPM <80/min. Blood pressure and pulse rate measurements were performed every 3 min for the anaesthesiologist to adjust the drug dose, but only drug dose and measurements at baseline and at 10, 20 and 30 min are given in Figure 1 and Table 2.

Hypertension was defined according to the Joint National Committee on Evaluation, Detection and Prevention of High Blood Pressure^17^ (our cut-off point for starting treatment was SBP >140 mm Hg), while SVT was considered when the patients suffered >100 BPM. Differences in the adverse events between the two drug formulations were also studied. Written informed consent was obtained from the participants or legally authorised representatives prior to conducting the study. Ethical approval was waived by the hospital’s ethics committee because the study was observational: the intervention was the standard of care for all patients.

American Society of Anesthesiologists Physical Status 1–4 (ASA-PS 1–4) patients, aged >18 years who suffered intra- and/or postoperative SVT and/or hypertension and treated with IV esmolol were included in this study. Patients with severe sepsis or septic shock and patients with normal blood pressure or hypotension who suffered perioperative tachycardia were excluded (fluid bolus was considered before b-blocker use in this group of patients), but if they remained tachycardic after having an adequate blood pressure increase, then they were enrolled in the study. Patients with atrial fibrillation or antihypertensive therapy were excluded as well. During the postoperative period, patients who suffered medium or severe pain (visual analogue scale (VAS) score >5) were also excluded.

To detect a 20% difference between the two groups of patients (assuming effect size = 0.8 and power 80%), we would need 52 patients (26 in each group) for our study to have adequate power. Instead, we included only 31 patients because the study ended earlier than expected (absence of one of the two drugs). The data presented were collected between May 2014 and July 2017. In Greek hospitals, there is only one GAD available for IV esmolol administration. Because both formulations (the BND and GAD) were available in the hospital, the anaesthesiologist on duty could use any of them (Breviblock Premixed Injection (esmolol HCI), Baxter Healthcare Corporation, Deerfield, IL, USA – (BND) – or Esmocard (Orpha-Devel Handels und Vertriebs GmbH Wintergasse 85/1B, A-3002 Purkersdorf, Austria) – (GAD)). Four different anaesthesiologists contributed with cases, while both formulations (BND and GAD) were available in the hospital only for the first 31 patients, which resulted in the early termination of the study. To minimise the investigators’ bias risk, the doctors were encouraged to use the two drugs alternately. Cases contributing to the study come from only one operating room unit (six operating rooms) and one recovery room.

According to a predetermined plan, the patients were categorised into two groups according to the medication they received (BND or GAD), into three groups according to the surgery risk (low, medium and high) and into two groups according to the time they received the b-blocker medication (intraoperatively vs postoperatively). The two main adverse events evaluated were hypotension (defined as SBP <90 mm Hg) and bradycardia (defined as BPM <50/min).

### Statistics

A statistical analysis was performed with SPSS version 22.0 software package (IBM SPSS Statistics for MAC, version 22.0, Armonk, NY, USA). Categorical variables were analysed using the *χ*2 test, while continuous variables were analysed using a *t* test for normally distributed data and Mann–Whitney test for skewed data. An analysis of variance (ANOVA) repeated measures analysis was used to investigate possible differences between time-related factors, while a Bonferroni correction was used for multiple comparisons statistical adjustment. Interpolation lines were used for time-related differences between the doses of the two drugs and the patient’s haemodynamics. A logistic regression analysis for potential confounders was performed with R version 3.6.2.^18^ The statistical tests were two-sided, and p < 0.05 was considered statistically significant.

### Results

Seventy-one patients were assessed for eligibility, and 40 patients were excluded from the study. Most of the excluded patients were treated with antihypertensives (22 patients), while six patients suffered from atrial fibrillation. Of the remaining 12 patients excluded from the study, four patients suffered septic shock, while in eight patients, blood loss was thought to be the cause of the tachycardia.

Esmolol was used in 31 patients (16 GAD and 15 BND). The patient’s baseline characteristics are presented in Table 1. The BND and GAD groups did not differ in sex (% male, 55% vs 58%), age (mean (standard deviation (SD)), 72 (15) vs 74 (16)), all comorbidities (mean (SD), 2.3 (1.2) vs 2.5 (1.3)) and ASA-PS classification (1/2/3/4), (3/8/2/2 vs 2/10/3/1, p = 0.82). The surgery risk did not differ between the two groups (low/medium/high) (BND vs GAD, 4/7/4 vs 4/7/5, p = 0.96). Postoperatively, 11/15 patients in the BND group versus 11/16 in the GAD group received esmolol, while the remaining patients (4/15 BND group and 5/16 GAD group) received the drug starting from the intraoperative period (p = 0.78). The mean fentanyl dose (mcg/h) used for perioperative pain was similar in the two groups (BND/GAD, mean (SD), 138.6 (32) vs 140 (39.4), p = 0.92), while five and seven patients in the BND and GAD groups, respectively, had an epidural catheter for perioperative pain management. There were no differences between the groups regarding the two indications for participation in the study. More specifically, 10 patients in the BND group and 12 patients in the GAD suffered tachycardia, while the remaining four patients in each group suffered from hypertension. One patient in the BND group suffered from both tachycardia and hypertension. The basic characteristics of the patients are also presented in Table 1.

**Table 1.** Patient’s baseline characteristics.

Group	Brand name drug	Generic analogue drug	p-value
Age (years)	72	74	NS
Sex (% male)	55	58	NS
Chronic obstructive pulmonary disease (pt no)	2	3	–
Coronary artery disease (pt no)	3	4	NS
Congestive heart failure (pt no)	4	5	NS
Peripheral vascular disease (pt no)	3	4	NS
Cerebrovascular disease (pt no)	3	3	NS
Chronic renal failure (pt no)	2	1	–
Chronic haemodialysis (pt no)	0	1	–
Malignancy (pt no)	3	4	NS
Diabetes (pt no)	3	3	NS
Tobacco use (pt no)	8	7	NS
All comorbidities (no per patient)	2.3	2.5	NS
ASA-PS classification (pt no)			
ASA I	3	2	–
ASA II	8	10	NS
ASA III	2	3	–
ASA IV	2	1	–
Surgery risk (pt no)			
Low	4	4	–
Median	7	7	–
High	4	5	NS
Surgery type (pt no)			
General surgery	8	8	NS
Orthopaedic	6	7	NS
Neurosurgery	1	1	NS
Perioperative fentanyl dose (mcg)	400	450	NS
Epidural analgesia (pt no)	5	7	NS
Adverse events (pt no)			
Nausea	3	4	NS
Vomiting	3	4	NS
Bradycardia	0	0	–
Hypotension	3	2	NS

NS: not significant statistical differences; pt no: patient number; ASA-PS: American Society of Anesthesiologists Physical Status.

There was a statistically significant difference between the groups in bolus (mg/kg) and continues (mg/kg/h) drug dose used (BND/GAD, mean (SD), 0.30 (0.1) vs 0.38 (0.1), p = 0.03 for bolus dose, and 0.22 (0.09) vs 0.29 (0.08) for continued dose at 10 min (p = 0.03), 0.19 (0.06) vs 0.24 (0.05) at 20 min (p = 0.01) and 0.14 (0.05) vs 0.18 (0.05) at 30 min (p = 0.02)) (Figure 1), but there were no time-related statistical significant differences in the reduction rates of the two drugs (p = 0.47, Figure 1). The differences in drug doses between the groups ranged between 21.25% (bolus dose) and 24.8% (10 min). Data for SBP, DBP, MBP and BPM for the two groups are presented in Table 2. There were no time-related statistically significant differences between the two groups in SBP (p = 0.36), DBP (p = 0.25), MBP (p = 0.09) and BPM (p = 0.49). The logistic regression analysis showed no potential confounders to be related with our results (p > 0.1 for the four different anaesthesiologists, comorbidities, ASA-PS classification, surgery risk and surgery type). There were no differences in the adverse events between the two groups of patients. Three patients in the BND and four in the GAD group presented nausea and vomiting. There were no episodes of bradycardia, while hypotension was reported in two and three patients in the GAD and BND groups, respectively.

**Table 2.** Systolic, diastolic, mean blood pressure and beats per minute measurements at baseline, 10, 20 and 30 min after esmolol use and time-related differences (generic analogue vs brand name drug).

			Group (mean, SD)		Time-related differences (generic vs brand) p-value
			Generic analogue drug	Brand name drug	
Systolic blood pressure (mm Hg)	Time (min)	Baseline	170.5 (12.9)	164.8 (14.9)	0.36
		10	144.2 (11.4)	138.0 (9.8)	
		20	133.1 (7.2)	128.4 (5.4)	
		30	130.3 (4.7)	129.2 (4.5)	
Diastolic blood pressure (mm Hg)		Baseline	114.5 (9.6)	111.7 (13.4)	0.25
		10	98.8 (11.0)	95.8 (11.6)	
		20	89.9* (12.4)	79.6* (10.6)	
		30	85.8** (6.4)	78.0** (8.6)	
Mean blood pressure (mm Hg)		Baseline	134.8 (9.6)	127.4 (12.3)	0.09
		10	112.8 (11.7)	111.8 (11.0)	
		20	108.3 (12.6)	103.4 (12.6)	
		30	108.1*** (8.2)	97.1*** (10.1)	
Beats per minute		Baseline	115.3 (5.3)	115.3 (4.8)	0.49
		10	103.3 (4.6)	102.8 (4.3)	
		20	91.6 (5.1)	89.6 (5.1)	
		30	81.0 (5.4)	79.2 (5.2)	

* p = 0.02; **p = 0.01; ***p = 0.003.

## Discussion

Most clinicians are repeatedly exposed to anecdotal evidence claiming that generic drugs are not as effective and/or safe as their branded counterparts.^14–16^ To date, 11 cross-over, randomised, controlled trials have evaluated the efficacy and safety of generic versus brand name b-blockers, all of which have looked at oral medications.^19–29^ More specifically, the main efficacy outcome that was evaluated was the SBP reduction from baseline to the end of follow-up. All the previous studies showed nonsignificant differences between the generic and brand name b-blockers when considering efficacy, bioequivalence and adverse events. To the best of our knowledge, our study is the first to evaluate the effectiveness and safety of perioperative IV generic esmolol use versus its branded analogue.

In our pilot study, higher doses of the GAD compared with the BND of IV esmolol were used to control the patient’s haemodynamics during the intra- and postoperative period, while the adverse events were similar in the two groups of patients (GAD vs BND group). Importantly, there were no significant time-related differences in the reduction rates of the two drugs, nor were there any time-related haemodynamic differences between the two groups. Our results show possible biodistribution, pharmacodynamics and/or pharmacokinetics discrepancies between the two drugs, but the optimal titration of the drug used (brand or generic) could effectively control the patient’s haemodynamics. In the context of cardiovascular diseases, two meta-analyses compared the outcomes of generic and brand name medications, which included the use of b-blockers, too. The first one was published in 2008 and showed no significant differences in terms of safety and tolerability between the drugs; this coincides with the results of our study.^30^ In the same meta-analysis, however, major questions were brought up regarding the clinical bioequivalence between the two types of drugs, which was also shown in our study in terms of biodistribution, pharmacodynamics and/or pharmacokinetics. A relatively recent meta-analysis including 53 trials and 2609 subjects and evaluating efficacy outcomes found no significant differences between generic and brand name cardiovascular drugs.^31^ Similarly, the risk of adverse events was comparable between generic and brand name medicines. It should be noted once again that all medicines were taken orally, while the meta-analysis included eight trials evaluating the differences in generic and branded b-blockers.

Our study has some limitations. According to our power analysis, a sample size of 52 patients would be needed for our study to have adequate power. Because the study terminated earlier, only 31 patients were included (from a point in time and beyond, only one of the two drugs was available in the hospital), meaning that it was underpowered. Furthermore, this was an observational study without any randomisation or blinded procedures. The observational design of our study and the limited number of patients could introduce both type I and type II errors in our data results. Because the control of the haemodynamic parameters was left to the anaesthesiologist on duty to determine, the apparent median standardisation (according to the patient’s baseline measurements while focusing SBP <140 mm Hg and BPM <80/min) makes interpretation of the dosing requirements difficult, and this is a limitation of our study as well. In the most resistant cases of hypertension and tachycardia, the best possible choice of the anaesthesiologist on duty would be the brand name preparation, and this could also introduce major bias in our study (type II error). The randomisation of the patients in the two groups would have solved part of the problem, but our study unfortunately was not randomised. The dose given in the GAD group was larger than in BND group already at baseline. The anaesthesiologists’ anticipation of the effect would be a possible explanation for that. However, even though not statistically significant, patients in the GAD had higher baseline systolic, diastolic and MBP measurements, which could have led the anaesthesiologists to use larger baseline doses. In fact, although there were no any time-related haemodynamic differences between the two groups, there were statistical significant differences at specific time points, while the systolic, diastolic and MBP measurements and BPM were almost always higher in the GAD group (Table 2).

Except from the observational design of our study, the small number of patients included and the possible biodistribution differences between generic and BND, some differences in the user’s characteristics could also explain our findings. Furthermore, the small drug dose differences depicted, although statistically significant, could be characterised as having minimal clinical significance (range between 21.25% (bolus dose) and 24.8% (10 min)) and possibly follows the principle accepted in Europe according to which the 90% CI of the ratios for AUC_0–t_ and C_max_ between the two preparations lie in the range 80%–125%. Even if bioequivalence discrepancies exist, optimal titration of the drug used (brand or generic) could effectively control the patient’s haemodynamics. The objectives of our pilot study will be answered much more confidently by conducting prospective, randomised controlled trials with sufficient validity; until then, the analysis of our data deserves attention.

## Conclusion

In this small pilot study, there was a difference in bolus and continuous drug dose between the BND and GAD of IV esmolol; in particular, smaller doses were given when the BND was used. However, there were no time-related significant differences in the reduction rates of the two drugs, nor were there any time-related haemodynamic differences between the two groups. Adverse events were also similar in both groups. According to our results, although some biodistribution/bioequivalence discrepancies between the two drugs are possible, optimal titration of the drug could effectively control the patient’s haemodynamics. Because this is an observational pilot study with an analysis of a limited number of patients, our results merit attention and future work.
